# Development of a nomogram predicting the probability of stone free rate in patients with ureteral stones eligible for semi-rigid primary laser uretero-litothripsy

**DOI:** 10.1007/s00345-021-03768-5

**Published:** 2021-06-26

**Authors:** Cosimo De Nunzio, Jamil Ghahhari, Riccardo Lombardo, Giorgio Ivan Russo, Ana Albano, Antonio Franco, Valeria Baldassarri, Antonio Nacchia, Juan Lopez, Pilar Luque, Maria Jose Ribal, Antonio Alcaraz, Andrea Tubaro

**Affiliations:** 1 Department of Urology, “Sant’Andrea” Hospital, “La Sapienza” University, Rome, Italy; 2 Hospital Clinic Barcelona, Barcelona, Spain

**Keywords:** Stone free, Ureterolitothripsy, Ureteral stones, Treatment

## Abstract

**Purpose:**

Few tools are available to predict uretero-lithotripsy outcomes in patients with ureteral stones. Aim of our study was to develop a nomogram predicting the probability of stone free rate in patients undergoing semi-rigid uretero-lithotripsy (ULT) for ureteral stones.

**Methods:**

From January 2014 onwards, patients undergoing semi-rigid Ho: YAG laser uretero-lithotripsy for ureteral stones were prospectively enrolled in two centers. Patients were preoperatively evaluated with accurate clinical history, urinalysis and renal function. Non-contrast CT was used to define number, location and length of the stones and eventually the presence of hydronephrosis. A nomogram was generated based on the logistic regression model used to predict ULT success.

**Results:**

Overall, 356 patients with mean age of 54 years (IQR 44/65) were enrolled. 285/356 (80%) patients were stone free at 1 month. On multivariate analysis single stone (OR 1.93, 95% CI 1.05–3.53, *p* = 0.034), stone size (OR 0.92, 95% CI 0.87–0.97, *p* = 0.005), distal position (OR 2.12, 95% CI 1.29–3.48, *p* = 0.003) and the absence of hydronephrosis (OR 2.02, 95% CI 1.08–3.78, *p* = 0.029) were predictors of success and these were used to develop a nomogram. The nomogram based on the model presented good discrimination (area under the curve [AUC]: 0.75), good calibration (Hosmer–Lemeshow test, *p* > 0.5) and a net benefit in the range of probabilities between 15 and 65%. Internal validation resulted in an AUC of 0.74.

**Conclusions:**

The implementation of our nomogram could better council patients before treatment and could be used to identify patients at risk of failure. External validation is warranted before its clinical implementation.

## Introduction

Ureteral stones are actually one of the most prevalent urological conditions accounting for 1% of emergency department visits [1]. If left untreated, they can often conduce to ureteral obstruction, kidney damage and/or in severe cases to the development of urinary sepsis [2, 3]. In the management of ureteral stones, treatment options include observation with medical expulsive therapy (MET), extracorporeal shock-wave lithotripsy (ESWL), uretero-lithotripsy (ULT) and laparoscopic or open uretero-lithotomy. Indications for active removal of ureteral calculi are persistent obstruction, renal insufficiency, persistent pain despite analgesic treatment and low likelihood of spontaneous stone passage [4]. Nowadays, most of the ULT are performed with Ho:YAG laser, however, recently several studies have demonstrated great outcomes using thulium laser [5].

ULT is currently an effective and safe procedure although not free from possible complications such as ureteral perforation, ureteral avulsion, acute sepsis, kidney injury and future need of further treatments [6–8]. Moreover, in 24–28% of the cases, patients may present residual fragments and the procedure failure rate/inability to access unstented ureter ranges from 0.7 to 7.7% [9–11].

In this clinical scenario, it appears therefore of great importance to identify those patients at high risk of treatment failure and to inform them of the possibility of multiple procedures to achieve a stone free status. In the past years, only one nomogram has been developed in a Japanese cohort to predict ULT outcomes [12]. The model has been validated by our group and has an accuracy of 0.67–0.74 on ROC analysis; however, several studies on predictive models suggest that they should be used in populations similar to the development cohort to improve discrimination and calibration of the model [10, 13].

With this knowledge in mind, aim of our study was therefore to develop a new nomogram predicting the probability of stone free rate in European countries, especially in the Mediterranean population, after semi-rigid ULT surgery (carried out using a Ho:YAG laser) for ureteral calculi diagnosed only by non-contrast CT.

## Materials and methods

### Study population

From January 2014 onwards, data from a consecutive series of patients undergoing semi-rigid Ho: YAG laser primary uretero-lithotripsy for ureteral stones were prospectively enrolled in two centers, at "Sant'Andrea" Hospital, "La Sapienza" University, Rome, Italy and at “Hospital Clinic Barcelona”, Barcelona, Spain. The study was approved by a local ethical committee and a specific informed consent was signed by all the patients. Study protocol was built in accordance with the principles of the declaration of Helsinki.

Age, gender and Body Mass Index (BMI), calculated as Kg/m^2^, were recorded from all patients.

Patients were preoperatively evaluated with accurate clinical history, blood sample including blood cell count, serum electrolytes, renal function (creatinine), uric acid, emo-coagulative evaluation, complete urine tests and urine cultures. The presence of pyuria was defined as at least ≥ 5 white blood cells/high power field on examination urinary sediment or urinary dipstick test positive for leucocyte esterase and/or nitrate.

Ureteral stones were diagnosed by non-contrast enhanced computer tomography (CT) that was used to define number, location and length of the stones and eventually the presence of hydronephrosis. For the variable number of stones, we have identified four categories as 1 stone, 2 stones, 3 stones, ≥ 4 stones. Stone length was defined as the maximum length on CT, whereas stone location was classified into ureteropelvic junction (UPJ), proximal ureter, middle ureter and distal ureter.

### Surgical technique

All patients underwent semi-rigid ULT under general or lumbar spinal anesthesia and procedure was carried out by expert surgeons, in two centers. ULT was performed using a 7,5 F semi-rigid ureteroscope using Road runner safe wire (Cook Medical, Bloomington, IN, USA) under fluoroscopy guidance. The energy source was a Ho:YAG laser ( Sphynx LISA laser, Katlenburg-Lindau Germany) using a 350 micron laser fibers employing 1 J and 10 Hz. All the stones were treated with a dusting technique and none of the stones was removed directly with basket or forceps. Any lithiasic residues have been removed through the use of forceps or baskets. Procedure was completed with the insertion of DJ ureteral stent to avoid stressful emergencies due to ureteral trauma, bleeding or residual fragments undiagnosed. All the patients with complications as low ureter compliance, ureteral perforation and ureteral stenosis were managed with ureteral stent for 4 weeks and second look intervention.

### Outcomes

Treatment efficacy and residual stones were evaluated at 1 month after surgery with non-contrast enhanced CT. Stone free was considered as no fragments detected on CT imaging. Patient with residual lithiasis underwent a second procedure such as ESWL, a second ULT, mini PCNL to obtain stone clearance.

### Statistical analysis

Statistical analysis was performed using the Statistical Package for the Social Sciences (SPSS v.24, IBM Corp., Armonk, NY, USA) and STATA software. Considering a stone free rate of 80% and supposing a AUC of 0.70 with an alpha value of 0.01 and a beta value of 0.99, the sample size calculated according to Obuchowski et al. [14] was 306. We estimated a 20% probability of drop-off with a resulting final sample size of 361 patients. Evaluation of data distribution using the Kolmogorov–Smirnov test showed a non-normal distribution of the study data set. Differences between groups of patients in medians for quantitative variables and differences in distributions for categorical variables were tested with the Kruskal–Wallis one-way analysis of variance and chi-squared test, respectively. All variables were assessed using univariate binary logistic regression for the prediction of stone free rate. The statistically significant variables were then entered into a multivariable age adjusted logistic regression model. Based on the multivariable model, a nomogram was developed. Receiver operator characteristic curves (ROC) were produced to evaluate the discrimination of the model. Calibration was assessed using the Hosmer–Lemeshow test (for this test, a *p* value < 0.05 indicates a poor agreement between predicted probabilities and observed outcome). Additionally, calibration plots were assessed, where the *x*-axis represents the predicted probability and the *y*-axis represents the actual observed accuracy of the model. Decision curves were generated to evaluate the net benefit of the model. Finally, a nomogram was developed based on the logistic regression model. A 200 bootstrap was used for internal validation. An alpha value of 5% was considered as the threshold for significance. Data are presented as median (range) and mean ± standard deviation (SD) and median with interquartile range (IQR).

## Results

Overall, 356 consecutive patients, including 242 (68%) males and 114 (32%) females, were prospectively enrolled in the study. Overall, in 12/356 patients, the procedure was not completed because of low ureter compliance, 4/356 patients presented a ureteral perforation, and 9/356 patients presented a ureteral stenosis. The characteristics of the study population are reported in Table 1.

**Table 1:** General cohort’s characteristics

	Overall	No stone free	Stone free	*p*
Number of patients	356	71/356 (20%)	285/356 (80%)	
Gender (male/female)	68%/32%	63%/33%	68%/22%	0.414
Median age (years)	54 (IQR:44/66)	55 (IQR:46/67)	54 (IQR:44/66)	0.768
Median length of the stone (mm)	8 (IQR:6/11)	10 (IQR:5/10)	8 (IQR:5/10)	0.001
Number of stones				
1	294/356 (82%)	54/71 (76%)	240/285 (85%)	0.001
2	46/356 (13%)	11/71 (16%)	31/285 (11%)	
3	8/356 (2%)	2/71 (3%)	6/285 (2%)	
4	8/356 (2%)	5/71 (5%)	3/285 (1%)	
Stone location				
UPJ	44/356 (12%)	18/71 (25%)	26/285 (9%)	0.001
Proximal ureter	61/356 (17%)	14/71 (19%)	47/285 (16%)	
Middle ureter	96/356 (27%)	25/71 (35%)	71/285 (24%)	
Distal ureter	155/356 (44%)	14/71 (21%)	141/285 (51%)	
Pyuria				
Yes	95/356 (27%)	27/71 (38%)	68/285 (24%)	0.008
Not	261/356 (73%)	44/71 (62%)	217/285 (76%)	
Hydronephrosis				
Yes	271/356 (76%)	57/71 (80%)	214/285 (75%)	0.001
Not	85/356 (24%)	14/71 (20%)	71/285 (25%)	
Stone density (HU)	993 (715–1218)	1019 (896–1321)	984 (834–1223)	0.234

285 (80%) patients were stone free (SF) at 1 month of follow-up, with no stones detected by CT scan. More specifically, SF was 70%, 80% and 85% for proximal, middle and distal ureteral stones, respectively. SF patients presented a lower median length of the stone (8 mm vs > 10 mm, *p* < 0.01). Moreover, SF patients presented more frequently a distal stone (51% vs 21%, *p* < 0.01) while presented less frequently hydronephrosis (75%vs 80%, < 0.01) and pyuria (24%vs38%, *p* < 0.01) when compared to NSF patients.

On multivariate analysis, single stone (OR 1.93, 95% CI 1.05–3.53, *p* = 0.034), stone size (OR 0.92, 95% CI 0.87–0.97, *p* = 0.005), distal position (OR 2.12, 95% CI 1.29–3.48, *p* = 0.003) and the absence of hydronephrosis (OR 2.02, 95% CI 1.08–3.78, *p* = 0.029) were predictors of stone free rate (Table 2).

**Table 2:** Multivariate analysis predicting the risk of stone free rate

	Multivariate	
	Odds ratio	*P*
Single stone	1.93 (1.05–3.53)	0.034
Stone size	0.92 (0.87–0.97)	0.005
Distal position	2.12 (1.29–3.48)	0.003
No hydronephrosis	2.02 (1.08–3.78)	0.029

**Fig. 1:**
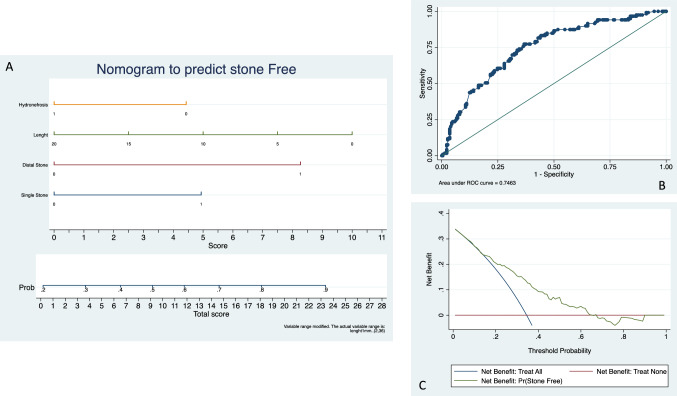
**A** Nomogram, **B** receiver operator characteristic curve, **C** decision curve analysis

The nomogram including the statistically significant variables to predict stone free rate presented a predictive accuracy of 0.75 on ROC analysis. The nomogram was well calibrated (*p* = 0.84) and presented a net benefit in the range of probability between 15 and 65% (Fig. 1). Internal validation with 200 bootstraps resulted in an area under the curve of 0.74.

## Discussion

In the present study, we successfully developed a nomogram to predict stone free rate in European multicentre cohort of patients. The model presented good discrimination properties (AUC = 0.75) and a clinical net benefit in the range of probabilities between 15 and 65%. As well the model was successfully internally validated (AUC = 0.74). According to our results single stone, small size, distal position and absence of hydronephrosis were independent predictors of stone free status.

In our multicentre cohort of patients, the overall SF rate was 80%. More specifically, SF was 70%, 80% and 85% for proximal, middle and distal ureteral stones, respectively. According to the available literature overall SF rates range from 60 to 100%. As well SF in proximal, middle and distal stones range from 51 to 75%, 56% to 88% and 81% to 93%, respectively. Our stone free rate is in line with the literature and confirm the internal validity of our results [10, 12, 15–18]. It is important to underline that no specific recommendation exists on imaging technique to use as follow-up after ULT [19, 20]. We decided to use non-contrast CT to reduce the biases linked to the interpretation of US or plain X-ray.

In our experience, the presence of large and/or multiple stones and/or with hydronephrosis was risk factors for residual fragments after ULT. These risk factors have been extensively confirmed by the available literature [17, 21]. El Nahas et al. showed that stones’ characteristics, proximal ureteral stone and inexperienced endourologic surgeon were unfavorable factors in ULT procedure [17]. As well Imamaura et al. and our validation of the Imamura nomogram confirmed the role of stone length, number of stones, stone location and pyuria as predictors of stone free rate [10, 12]. Other important predictors of SF status include surgeon experience and stone impaction [21]. Leijte et al. in their study on ULT predictive factors confirmed that surgeon experience is essential for a successful and safe procedure [21]. To exclude this possible bias, in our study, we decided to include only procedures performed by expert endo-urological surgeons with more than 10 years of experience and at least 100 procedures a year.

A clear definition of stone impaction on CT is still not available. Tran et al. recently evaluated the possible role of proximal and distal ureter HU on CT to predict stone impaction with promising results. To avoid possible biases on the definition, we decided not to evaluate this aspect in the present study; however, a study on the subject is ongoing and preliminary results have been published recently in an abstract [22, 23].

In the past years, thousands of predictive models have been developed to predict outcomes after surgery. In urology, several predictive models have been developed both in oncological and stones field; however, only one model demonstrates to predict stone free status after ULT [10, 12, 24–31]. The Japanese group of Imamura was the first to publish a nomogram to predict ULT efficacy [12]. The model included stone length, number, location and the presence of pyuria. It presented a predictive accuracy of 0.74 which was confirmed by the external validation by our group (AUC = 0.67, *p* < 0.01) [10, 12]. After long years of developing nomograms, it became quite evident that general characteristics of the population clearly influence the discrimination and calibration abilities of the model [13]. In our validation of the Imamura nomogram, the nomogram showed fair discrimination abilities and poor calibration [10]. With this knowledge in mind, we decided to develop a nomogram to use in a south Mediterranean population. The Imamura model is lacking important statistical data regarding clinical net benefit which is of great importance when developing a predictive model. Our model presented good discrimination abilities and a clinical net benefit which represents an important factor to consider when evaluating predictive models.

In the present study, all patients underwent ULT using the sphynx holmium laser which is limited by a maximum power of 10j and does not allow changes in pulse rate. Nowadays, laser technology has been improved with different effects as Moses, vapor tunnel, Bubble blast and Virtual basket [32]. As well different fibers diameters are available which enable the surgeon to decide whether to deliver more power or precision. Moreover, the use of a stone cone may improve the stone free rate limiting the stone migration episode. Lastly, different baskets are available in the market which can improve stone free rate [33]. Prediction models in based on these different new technologies are not available at the moment and may represent a future area of research.

In the past years, clinicians have focused on risk-based or personalized medicine. Identification of patients at high risk of treatment failure can be based on a single or a combination of multiple risk factors. The prevailing thought is that combining predictors into a predictive model allows for a better risk assessment and patient selection than single predictors or tests. The predictive model has the advantage of an accurate planning of the intervention and allow for an individualized counseling of the patient. When evaluating a model, it is important to consider discrimination, calibration, clinical benefit, internal validity, model presentation and external validity [13]. Our model has the merit of presenting good discrimination abilities, excellent calibration, good clinical benefit, internal validation and a user-friendly model presentation.

**Fig. 2:**
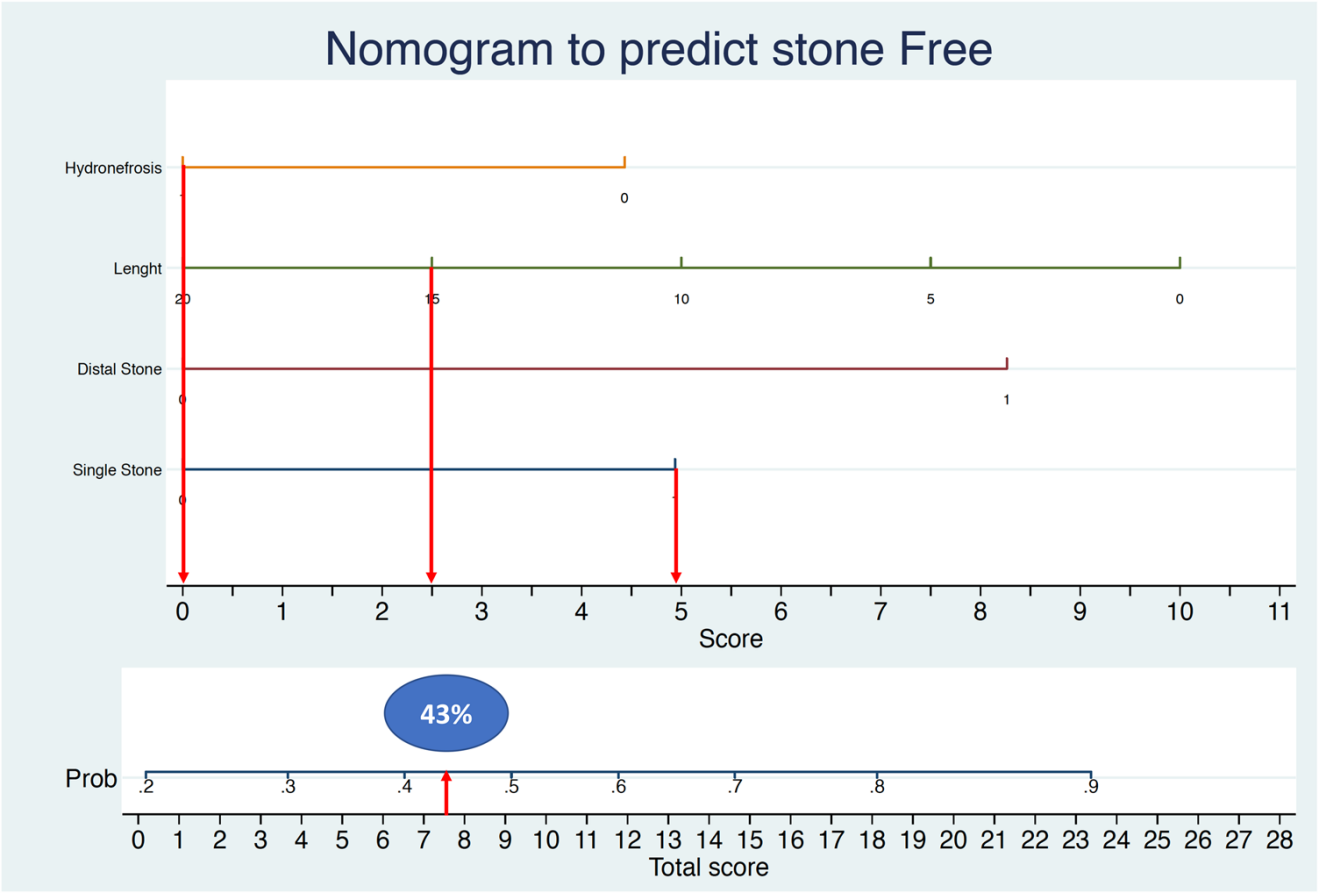
We present the case of a patient with a 15 mm single stone located proximally with hydronephrosis. To obtain the nomogram-predicted probability of stone free, locate the patient’s variable values at each axis. Draw a vertical line to the ‘Points’ axis to determine how many points are attributed for each variable value (0 Points for hydronephrosis, 2,5 points for stone size, 5 points for a single stone and 0 points for a proximal stone). Sum the points for all variables and locate the sum on the ‘Total points’ line (total points 7, 5 points). Draw a vertical line from the total point’s axis toward the ‘Probability of stone free’ axis to determine the patients’ stone free (stone free probability 43%)

The present model has important clinical implications in the preoperative counseling of the patient. A patient with a low probability of stone free rate (Fig. 2) may decide to perform an alternative less invasive treatment as ESWL or a more invasive one like miniPCNL. Recently, Dasgupta and coworkers have demonstrated the non-inferiority of ESWL vs URS in treating ureteral stones [34]. Moreover, in an era of common litigation after surgery, a patient which is aware of a low probability of success or the need of multiple subsequent procedures (ESWL or a new ULT) will accept more easily an un-successful outcome. The clinical impact of our nomogram in patients’ decision-making is still to be determined and a prospective study is ongoing in our center. In our experience, no correlation between complications and preoperative characteristics was found (data not shown). However, in our study, only 25 complications were recorded and therefore no definitive conclusion can be made. A large multicenter prospective study is ongoing to better elucidate this aspect. The clinical impact of our nomogram in patients’ decision-making is still to be determined and a prospective study is ongoing in our center.

We have to acknowledge some limitations to the present study. The model needs external validation before clinical implementation and possibly using an app model presentation. However, a study to develop a mobile-based app to be externally validated is ongoing and results will be soon available. ‘A possible limitation of our study is that in case of stone retropulsion flexible ureteroscope was not used, particularly our study was focused only on semi-rigid ureteroscopy. Nomogram on flexible ureteroscopy are available in the literature and include other variables which not apply to semi-rigid ureteroscopy [35].’ Our study was performed in a south Mediterranean cohort of patients undergoing ULT in expert hands and therefore our results clearly depend on the enrolled population. Furthermore, our data are strictly dependent on characteristics of stone size, location and number, which can clearly differ from other clinical scenarios. Another possible limitation is the lack of data on patients which performed concomitant flexible ULT; however, flexible ureteroscopes are not available in all settings and therefore we preferred to exclude these patients. Another study is ongoing to analyze predictive factors in this particular population. A possible limitation of our nomogram is the lack of power to perform an analysis on complications; however, a large multicenter study to investigate predictors of complications in patients undergoing uretero-lithotripsy is ongoing. As well, stone analysis was not performed in our study. Despite the limitations, the proposed semi-rigid UTL-predictive model, can prove to be an excellent tool to assess with patient treatment outcomes and to bring him closer to the so-called "tailored medicine", facilitating counseling and responding more to patient’s needs in terms of clarity of results and therapeutic strategies. In the field of ULT procedures, this model was the first to be evaluated with decision curve analysis that calculates clinical “net benefit” of nomogram in comparison to default strategy. External validation of this new predictive model is required before its implementation in clinical practice. However, notwithstanding all these limitations, if externally validated our nomogram could represent and easy tool to council patients with ureteral stones and to possible identify patients at major risk of residual fragments who might need further treatments.

## Conclusion

In our study, we developed a clinical nomogram to predict stone free rate (SFR) in patients undergoing uretero-lithotripsy for ureteral stones. This nomogram showed reasonable accuracy in predicting SFR; if externally validated, our nomogram could be used in clinical practice to council patients and to identify patients at major risk of residual fragments needing subsequent treatments.

## Data Availability

Data and material are available upon request.
